# Clinical burden of *Acinetobacter baumannii*, including carbapenem-resistant *A. baumannii*, in hospitalized adult patients in the USA between 2018 and 2022

**DOI:** 10.1186/s12879-025-10749-1

**Published:** 2025-04-17

**Authors:** Thomas P. Lodise, Sean T. Nguyen, Caroline Margiotta, Bin Cai

**Affiliations:** 1 Albany College of Pharmacy and Health Sciences, Albany, NY USA; 2 Present Address: Shionogi Inc, 400 Campus Drive Florham Park, Florham Park, NJ 07932 USA; 3 Genesis Research, Hoboken, NJ USA

**Keywords:** *Acinetobacter baumannii*, Carbapenem resistance, Epidemiology, Length of stay, Mortality

## Abstract

**Background:**

Epidemiological data for United States (US) hospitals regarding the burden of *Acinetobacter baumannii* and carbapenem-resistant *A. baumannii* (CRAB) are scarce; thus, this study aimed to describe the incidence of *A. baumannii* and CRAB across US hospitals between January 1, 2018 and December 31, 2022.

**Methods:**

This was a retrospective cohort study of hospitalized patients with microbiology data from the PINC AI™ Database. Incidence rates of *A. baumannii* and CRAB (January 1, 2018 – December 31, 2022) were determined across US hospitals in each census region. Incidence rates of *A. baumannii* and CRAB were determined at the hospitalization encounter and individual levels. Presence of CRAB was based on non-susceptibility to either doripenem, imipenem, or meropenem. Patient demographics, comorbidities, outcomes, including in-hospital mortality, were compared between patients with CRAB and carbapenem-susceptible *A. baumannii* (CSAB) at the hospitalization encounter level.

**Results:**

During the study period, 7,270 hospitalization encounters with ≥ 1 *A. baumannii* clinical cultures were identified. The overall *A. baumannii* incidence rate was 1.19 cases per 100 hospitalization encounters and 1.33 cases per 100 unique patients. For CRAB, a total of 2,708 hospitalization encounters were identified, and incidence rate was 0.44 cases per 100 hospitalization encounters. The West South Central, East North Central, and East South Central regions had the highest CRAB incidence rates (0.78, 0.67, and 0.63 cases per 100 hospitalization encounters, respectively). Compared with CSAB, patients with CRAB had significantly more positive cultures with *A. baumannii* (20.9% vs. 10.0%, respectively, *P* < 0.0001) and higher prevalence of other Gram-negative pathogens in any clinical culture site within ± 3 days of the index *A. baumannii* clinical culture (47.2% vs. 42.9%, respectively, *P* = 0.0004). Patients with CRAB had higher incidences of in-hospital mortality vs. patients with CSAB (20.5% vs. 11.3%, respectively, *P* < 0.0001).

**Conclusions:**

Presence of *A. baumannii* was identified on a clinical culture in 1% of adult hospitalizations in this multicenter US study. Over a third of *A. baumannii* hospitalization encounters were CRAB, with the highest incidence rates per 100 hospitalization encounters observed in the most central US regions. Clinicians should consider *A. baumannii* as a potential pathogen in patients in regions with an increasing incidence rate of *A. baumannii*.

**Supplementary Information:**

The online version contains supplementary material available at 10.1186/s12879-025-10749-1.

## Background

*Acinetobacter baumannii* is a problematic pathogen worldwide [1, 2]. In the USA, it is a major cause of healthcare-associated infections, representing approximately 2% of all healthcare-associated infections, and the ability of the organism to survive in hospital environments and colonized patients for extended periods of time have contributed to the increased incidence of outbreaks caused by the organism [3–5]. In the recent report issued by the National Healthcare Safety Network, *A. baumannii* was found to be one of the leading causes of ventilator-associated pneumonia, central line-associated bloodstream infection, and urinary tract infections in hospitalized adult patients [6].

Management of patients with *A. baumannii* infections is challenging due to the pathogen’s high capacity for antimicrobial resistance through multiple resistance mechanisms [7–9]. Carbapenem resistance in *A. baumannii* (CRAB) is now commonplace [2, 9–12] and the Antimicrobial Resistance Laboratory Network (AR Lab Network) recently reported that carbapenemase genes were detected in 83% of CRAB isolates in 2019 [13]. The US Centers for Disease Control and Prevention (CDC) estimated that there were approximately 8,500 cases of carbapenem-resistant (CR) *A. baumannii* (CRAB) per year and 700 associated deaths, with the prevalence of CRAB being reported to be considerably higher in other parts of the world [14]. According to their latest report published in 2022, the CDC estimated 6000 and 7500 cases of CRAB with approximately 500 and 700 deaths, respectively, for the years 2019 and 2020 [15]. Both the CDC and the World Health Organization (WHO) have designated CRAB as a critical pathogen, for which new antibiotics are urgently needed [1, 14]. It has been recognized that carbapenem resistance in *A. baumannii* is significantly associated with increased mortality risk (i.e., up to 55% all-cause mortality [11, 16] and 16% attributable mortality [17] and prolonged hospitalization [18, 19], in both randomized controlled studies and retrospective observational studies [11, 18, 20, 21]. The increased risk of mortality in CRAB infection may partially be the result of inadequate pathogen clearance due to lack of active antibiotics [18]. However, a recent observational study conducted in India showed that, despite the administration of recommended antibiotics with in vitro activity in combination therapy to patients with CRAB, mortality rate may be as high as > 60% in the presence of clinical risk factors (e.g., shock) [22]. Despite the recognition by the CDC and WHO of CRAB as an “urgent threat” globally [1, 3, 14], only limited data are available on the incidence rates of *A. baumannii* and CRAB across US hospitals. Given this critical gap in the literature, the objective of this multicenter observational study was to gain a better understanding of the epidemiology of *A. baumannii* and CRAB among hospitalized adult patients in the USA. Additionally, outcome analyses were performed to benchmark the clinical outcomes observed in patients with CRAB and CSAB on a clinical culture.

## Methods

### Study design and population

The population included in this study were adult hospitalized patients (aged ≥ 18 years) who had microbiology data and presence of *A. baumannii* on a clinical culture between January 1, 2018 and December 31, 2022 in the US PINC AI™ electronic healthcare database (Premier Inc., Charlotte, NC, USA; formerly known as Premier Healthcare Database) [23]. The US PINC AI™ electronic healthcare database is a hospital-based, service-level, all-payer database that currently contains information from more than 1,400 geographically diverse non-profit, non-governmental, community and teaching hospitals and health systems from rural and urban areas. It is updated weekly and represents around 25% of annual US inpatient admissions with clinical information. A subset of over 516 hospitals in the US PINC AI™ provided microbiology laboratory data at various times from 2009 to June 2024 [23]. During the study period of January 2018 to December 2022, 338 hospitals reported clinical and microbiological information in the US PINC AI™ electronic healthcare database. Information in the database is de-identified, making it fully compliant with the Health Insurance Portability and Accountability Act and exempting it from institutional review board or ethics committee approval.

### Hospital- and patient-level covariates

Hospital level covariates included USA geographic region and US Census-defined division (Northeast: New England, Middle Atlantic; Midwest: East North Central, West North Central; South: South Atlantic, East South Central, West South Central; and West: Mountain, Pacific), hospital size (bed capacity; <100, 100–199, 200–299, 300–399, 400–499, ≥ 500) and urban (population density of core census group and surrounding census blocks of 1000 people/square mile and 500 people/square mile, respectively) vs. rural (any area not defined as urban) area [23]. Patient-level covariates included age, sex, race, Charlson Comorbidity Index (CCI) score [24], comorbidities, admission type (emergency [generally through the emergency room], urgent [generally to the first available and suitable accommodation], trauma center, elective, or other/unknown).

### Microbiology data

Microbiology data analyzed included time between day of *A. baumannii* culture collection and admission day, the number of positive *A. baumannii* clinical cultures, clinical culture site (i.e., respiratory, blood, urinary, wound, fluid/serum, lesion/abscess/ulcer, and other), carbapenem resistance status (CR or carbapenem susceptible [CS]), and the presence of other Gram-negative pathogens in a clinical culture (any culture site or same culture site) within ± 3 days of the index *A. baumannii* clinical culture. Presence of CRAB was based on the presence of a non-susceptible (intermediate or resistant) test result to either doripenem, imipenem, or meropenem. Similarly, other Gram-negative pathogens identified on clinical cultures ± 3 days of the index *A. baumannii* clinical culture were classified as carbapenem resistant if there was a non-susceptible (intermediate or resistant) test result to doripenem, imipenem, meropenem, or ertapenem (ertapenem susceptibility results were only used to identify carbapenem-resistant Enterobacterales [CRE]). Patients were considered to have CRAB if any of their *A. baumannii* isolates were CR. For the purposes of analyses, the collection day of their first CRAB clinical culture following their hospital admission was considered their index CRAB culture. Among patients with clinical cultures for carbapenem-susceptible *A. baumannii* (CSAB) only, the collection day of their first CSAB clinical culture following their hospital admission was considered their index CSAB culture. Readmissions due to *A. baumannii*, either CRAB or CSAB, were not analyzed.

### Outcomes

The primary outcomes assessed in the study included hospital discharge destination (in-hospital death, discharged to hospice, home, transferred to another healthcare facility, and other), 14- and 30-day in-hospital mortality, total hospital length of stay (LOS), and infection-associated hospital LOS (i.e., hospital LOS from index *A. baumannii* culture collection day to death or hospital discharge). The ICU infection-associated LOS was also determined for patients who were in the ICU from the day of the index culture day until their discharge or death. Furthermore, ICU infection-associated LOS was evaluated for patients admitted to the ICU on or after the index *A. baumannii* culture collection day.

### Statistics

Incidence rates of *A. baumannii*, CRAB, and CSAB were determined at the hospitalization encounter (cases per 100 hospitalization encounters) and unique patient (cases per 100 unique patients) levels using descriptive statistics during the study period and by year. Incidence rates of *A. baumannii*, CRAB, and CSAB on both the hospitalization encounter and unique patient levels were determined across the four geographic regions and US Census-defined divisions. Incidence rates of *A. baumannii*, CRAB, and CSAB were also stratified by presence of other Gram-negative pathogens in a clinical culture (any culture site or same culture site) within ± 3 days of the index *A. baumannii* clinical culture. Baseline hospital- and patient-level covariates were compared between patients with CRAB and CSAB on the hospital encounter level. Bivariate comparisons between patients with CRAB vs. CSAB were conducted using a *X*^2^ test for categorical variables and a Wilcoxon rank sum test for continuous variables. Analyses were performed using SAS 9.4 (Cary, NC, USA).

## Results

### *A. baumannii* incidence rates on the hospitalization encounter and unique patient levels

**Fig. 1:**
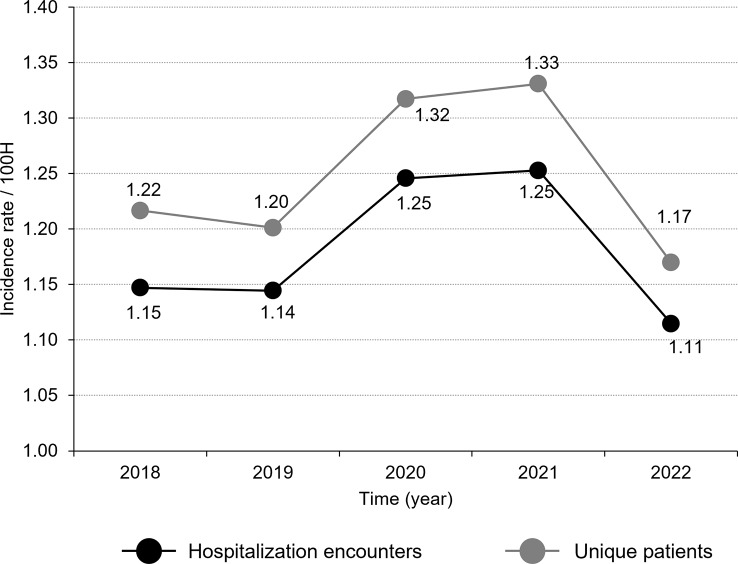
*Acinetobacter baumannii* incidence rate, defined as number of cases per 100 hospitalization encounters, by year. Incidence rate by hospitalization encounters is defined as the proportion of cases with a positive *A. baumannii* clinical culture during any hospitalization period per 100 hospitalization encounters. Incidence rate by unique patients is defined as the proportion of individual patients with a positive *A. baumannii* clinical culture across all hospitalization periods per 100 hospitalization encounters

During the study period, a total of 314 of 338 hospitals with microbiology data reported *A. baumannii* and there were 7,270 hospitalization encounters with at least one positive clinical culture for *A. baumannii*. The overall incidence rates of *A. baumannii* were 1.19 cases per 100 hospitalization encounters and 1.33 cases per 100 unique patients. The rate of *A. baumannii* cases per 100 hospitalized patients was higher than the rate of *A. baumannii* cases per 100 hospitalization encounters because patients with hospitalizations in different years were counted only once. There was a slight increase in the incidence rate of *A. baumannii* cases per 100 hospitalization encounters between 2018 and 2021 with a subsequent decline in 2022. The annual rate of *A. baumannii* cases per 100 unique hospitalized patients over the study period trended in a similar fashion (Fig. 1).

**Fig. 2:**
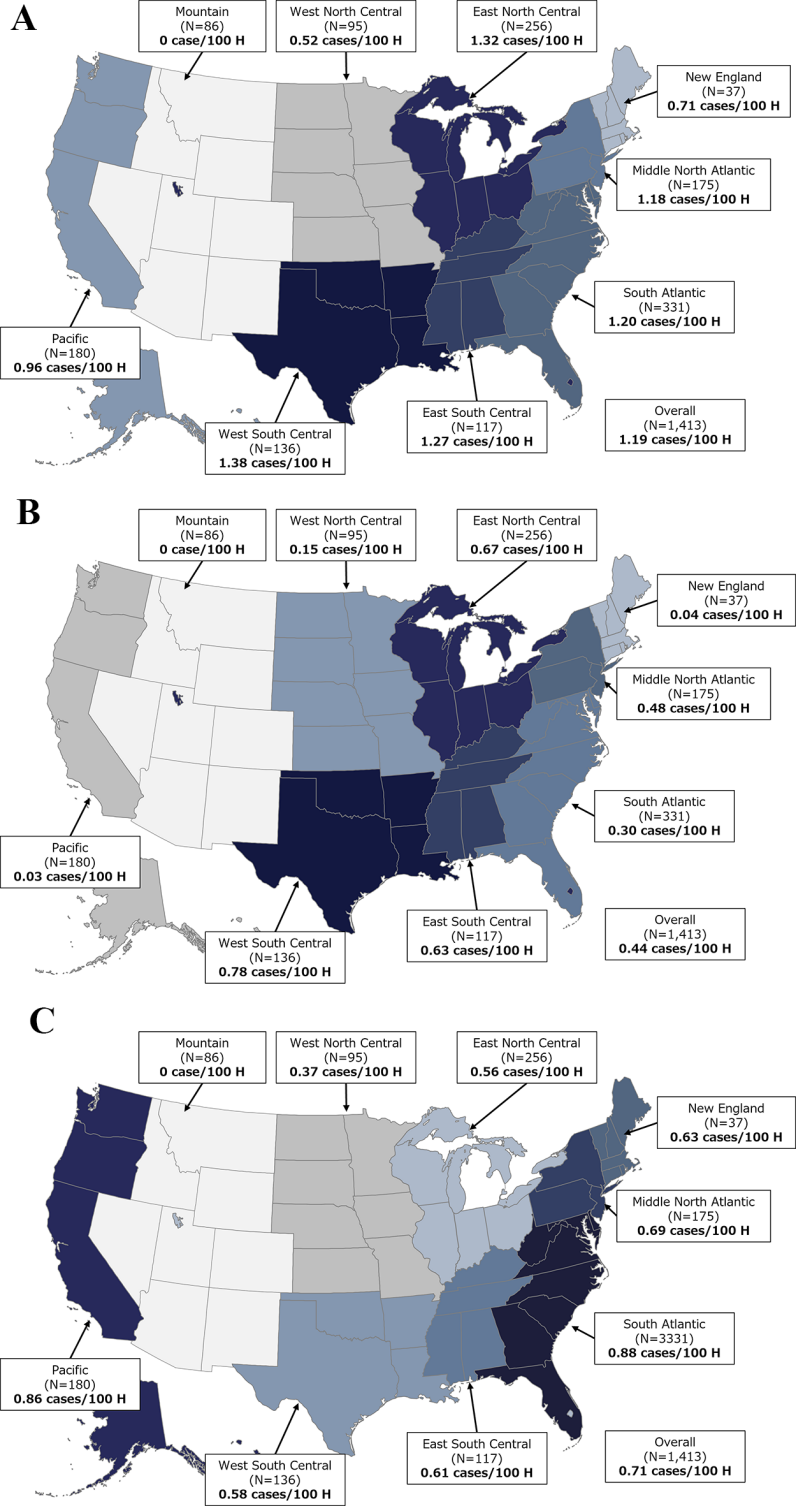
Incidence rates for (**A**) *Acinetobacter baumannii*, (**B**) CRAB, and (**C**) carbapenem-susceptible *A. baumannii.* CRAB, carbapenem-resistant *A. baumannii*; H, hospitalization encounter. The deeper/darker colors represent higher incidence rates. Number of institutions: Overall: 1,413; East North Central: 256; East South Central: 117; Middle Atlantic: 175; Mountain: 86; New England: 37; Pacific: 180; South Atlantic: 331; West North Central: 95; West South Central: 136. Number of institutions with microbiology results: Overall: 338; East North Central: 51; East South Central: 30; Middle Atlantic: 28; Mountain: 0; New England: 12; Pacific: 17; South Atlantic: 91; West North Central: 32; West South Central: 53. Number of hospitalization encounters with microbiology results: Overall: 613,244; East North Central: 97,596; East South Central: 61,575; Middle Atlantic: 72,435; Mountain: 0; New England: 30,930; Pacific: 11,937; South Atlantic: 244,755; West North Central: 26,874; West South Central: 67,142

Across the census regions, the West South Central region had the highest incidence rate of *A. baumannii* cases per 100 hospitalization encounters, followed by East North Central and East South Central regions (1.38, 1.32, and 1.27 cases per 100 hospitalization encounters, respectively) (Fig. 2A). There was an increasing trend in the incidence rate of patients with another Gram-negative pathogen in any site within ± 3 days of the index *A. baumannii* culture between 2018 and 2021 (Supplementary Table 1).

### CRAB and CSAB incidence rates on the hospitalization encounter and unique patient levels

**Fig. 3:**
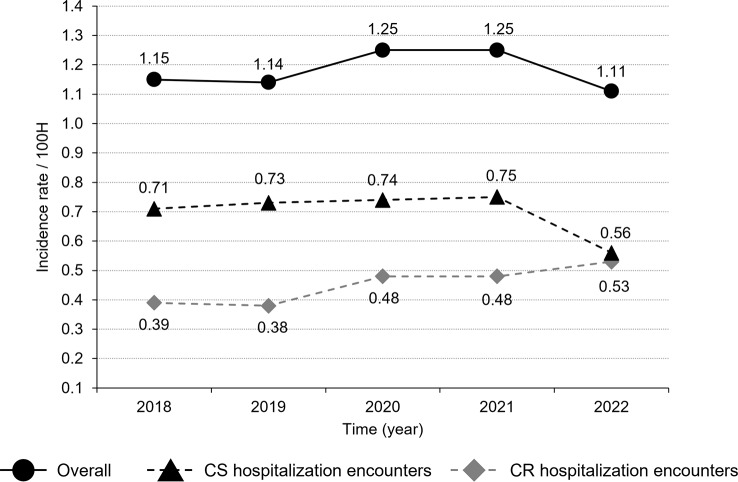
CRAB and carbapenem-susceptible *Acinetobacter baumannii* incidence rates per 100 hospitalization encounters. CR, carbapenem resistant; CRAB, carbapenem-resistant *A. baumannii*; CS, carbapenem-susceptible *A. baumannii*; H, hospitalization encounter. Incidence rate by hospitalization encounters is defined as the proportion of cases with a positive *A. baumannii* clinical culture during any hospitalization period per 100 hospitalization encounters

Carbapenem susceptibility data were available for 7,078 hospitalization encounters and it was unknown for 192 hospitalization encounters. During the study period, 37.2% of *A. baumannii* were CRAB (based on cultures that were solely CR or that were a mixture of CR and CS/carbapenem susceptibility unknown). The overall CRAB incidence rate was 0.44 cases per 100 hospitalization encounters. Between 2018 and 2022, annual incidence rates increased for CRAB, particularly between 2020 and 2022. Incidence rates for CS *A. baumannii* remained steady between 2018 and 2021, with a drop in 2022 (Fig. 3).

The highest CRAB incidence rates per 100 hospitalization encounters were in West South Central (i.e., 0.78 cases per 100 hospitalization encounters) and East North Central (i.e., 0.67 cases per 100 hospitalization encounters) regions (Fig. 2B). The Pacific region had the lowest CRAB rates (i.e., 0.03 cases per 100 hospitalization encounters; Fig. 2B). For CS *A. baumannii*, the highest rate per 100 hospitalization encounters was seen in the South Atlantic region followed by the Pacific region (i.e., 0.88 and 0.86 per 100 hospitalization encounters, respectively) (Fig. 2C).

### Comparison of baseline hospital- and patient-level covariates between CRAB and CSAB patients on the hospitalization encounter level

Comparison of hospital- and patient-level covariates at the hospital encounter level between patients with CRAB and CSAB are shown in Table 1. On the hospitalization encounter level, most patients with CRAB and CSAB were from hospitals with ≥ 500 beds (46.2% vs. 45.9%, respectively). Patients with CSAB were more frequently found in urban hospitals than patients with CRAB (84.9% vs. 79.3%, respectively, *P* < 0.0001) (Table 1). Patients with CRAB were significantly more frequently hospitalized in the East North Central, East South Central, West South Central census regions, while patients with CSAB were significantly more frequently admitted in New England and South Atlantic census regions (Table 1; *P* < 0.0001). Over 60% of patients with CRAB or CSAB were male. Patients with CRAB were significantly older than those with CSAB (64.0 median years vs. 60.0 median years, respectively, *P* < 0.0001). The median CCI on admission was 3.0 for both CRAB (IQR: 2.0–5.0) and CSAB (IQR: 1.0–5.0). The most frequent comorbidities in both groups were renal disease, diabetes with chronic complications, diabetes without chronic complications, chronic pulmonary disease, and congestive heart failure. Most comorbidities were more frequent in patients with CRAB than in those with CSAB (Table 1).

**Table 1:** Baseline hospital- and patient-level covariates in CRAB and CSAB patients at the hospitalization encounter level

Characteristics	CR *A. baumannii*^1^	CS *A. baumannii*^2^	*P* value
**Number of encounters**	2,708	4,370	
**Hospital bed size**,** n (%)**			
< 100	98 (3.6)	146 (3.3)	< 0.0001
100–199	250 (9.2)	479 (11.0)	
200–299	337 (12.4)	625 (14.3)	
300–399	635 (23.4)	680 (15.6)	
400–499	138 (5.1)	432 (9.9)	
≥ 500	1,250 (46.2)	2,008 (45.9)	
**Urbanicity**,** n (%)**			
Urban	2,147 (79.3)	3,710 (84.9)	< 0.0001
Rural	561 (20.7)	660 (15.1)	
**Census division**,** n (%)**			
New England	12 (0.4)	195 (4.5)	< 0.0001
Middle Atlantic	348 (12.9)	498 (11.4)	
South Atlantic	741 (27.4)	2,159 (49.4)	
East North Central	651 (24.0)	551 (12.6)	
West North Central	40 (1.5)	100 (2.3)	
East South Central	390 (14.4)	373 (8.5)	
West South Central	522 (19.3)	391 (8.9)	
Pacific	4 (0.1)	103 (2.4)	
**Admission information**			
**Admission type**,** ungrouped**,** n (%)**			
Elective	168 (6.2)	235 (5.4)	< 0.0001
Emergency	2,184 (80.6)	3,479 (79.6)	
Urgent	327 (12.1)	466 (10.7)	
Trauma center	29 (1.1)	188 (4.3)	
Information not available	0 (0.0)	2 (0.0)	
**Admission type**,** grouped**,** n (%)**			
Scheduled admission (elective)	168 (6.2)	235 (5.4)	0.1871
Emergency, trauma center, urgent	2,540 (93.8)	4,133 (94.6)	
Missing	0 (0.0)	2 (0.0)	
**Demographic characteristics**			
**Sex**,** n (%)**			
Female	1,045 (38.6)	1,713 (39.2)	0.6092
Male	1,663 (61.4)	2,657 (60.8)	
**Age**,** continuous**,** years**			
Median (IQR)	64.0 (54.0–73.0)	60.0 (48.0–71.0)	< 0.0001
Min–max	18.0–89.0	18.0–89.0	
**Age**,** categorical**,** n (%)**			
18–35 years	158 (5.8)	501 (11.5)	< 0.0001
36–55 years	606 (22.4)	1,179 (27.0)	
56–75 years	1,415 (52.3)	1,943 (44.5)	
≥ 76 years	529 (19.5)	747 (17.1)	
**Race**,** n (%)**			
White	1,761 (65.0)	2,972 (68.0)	< 0.0001
Black	708 (26.1)	923 (21.1)	
Other	239 (8.8)	475 (10.9)	
**Clinical characteristics**			
**CCI– On admission**			
Median (IQR)	3.0 (2.0–5.0)	3.0 (1.0–5.0)	< 0.0001
Min–max	0.0–17.0	0.0–19.0	
**Comorbidities on admission**,** n (%)**,** not mutually exclusive**			
Myocardial infarction	98 (3.6)	180 (4.1)	0.2925
Congestive heart failure	925 (34.2)	1,172 (26.8)	< 0.0001
Peripheral vascular disease	247 (9.1)	381 (8.7)	0.5626
Cerebrovascular disease	91 (3.4)	232 (5.3)	0.0001
Dementia	358 (13.2)	241 (5.5)	< 0.0001
Chronic pulmonary disease	973 (35.9)	1,404 (32.1)	0.0010
Rheumatologic disease	102 (3.8)	160 (3.7)	0.8196
Peptic ulcer disease	43 (1.6)	91 (2.1)	0.1379
Mild liver disease	245 (9.0)	395 (9.0)	0.9905
Diabetes without chronic complications	911 (33.6)	1,196 (27.4)	< 0.0001
Diabetes with chronic complications	972 (35.9)	1,288 (29.5)	< 0.0001
Hemiplegia or paraplegia	568 (21.0)	397 (9.1)	< 0.0001
Renal disease	982 (36.3)	1,260 (28.8)	< 0.0001
Any malignancy	163 (6.0)	419 (9.6)	< 0.0001
Moderate or severe liver disease	63 (2.3)	113 (2.6)	0.4958
Metastatic solid tumor	71 (2.6)	199 (4.6)	< 0.0001
AIDS/HIV	28 (1.0)	39 (0.9)	0.5501

AIDS, acquired immunodeficiency syndrome; CCI, Charlson Comorbidity Index; CR, carbapenem resistant; CS, carbapenem susceptible; HIV, human immunodeficiency virus; IQR, inter-quartile range; SD, standard deviation

^1^CR *A. baumannii* includes encounters where all cultures were CR, cultures were a mix of CR and CS, or cultures were a mix of CR and with unknown susceptibility

^2^CS *A. baumannii* includes encounters where all cultures were CS, or cultures were a mix of CS and with unknown susceptibility

### Comparison of microbiologic characteristics between CRAB and CSAB patients on the hospitalization encounter level

Comparison of microbiologic characteristics of the index *A. baumannii* clinical culture between CRAB and CSAB patients on the hospitalization encounter level is shown in Table 2. The proportion of patients with an *A. baumannii* index culture at admission was significantly lower for those with CRAB vs. CSAB (51.2% vs. 61.6%, respectively, *P* < 0.0001). Although the measure of central tendency (i.e., median) was identical between groups, the median (IQR) time, measured in days, between hospital admission and index culture day was found to be significantly different between CRAB and CSAB patients (2 (1–8) days vs. 2 (1–5) days, respectively, *P* < 0.0001). A higher percentage of patients with CRAB were in the ICU on the index culture collection day compared with patients with CSAB (43.0% vs. 38.1%, respectively, *P* < 0.0001). Patients with CRAB were less likely than patients with CSAB to have only one culture site (89.7% vs. 96.6%, respectively, *P* < 0.0001), although the presence of > 1 culture sites was low in both groups (10.3% vs. 3.4%, respectively). The most common culture site was the respiratory tract for both CRAB and CSAB groups (39.4% and 30.5%, respectively), followed by wound (34.1% and 29.2%, respectively) and urine (10.4% and 15.8%, respectively). Presence of other Gram-negative infections in any culture site within ± 3 days of the index *A. baumannii* clinical culture was significantly more common in patients with CRAB compared with patients with CSAB (47.2% vs. 42.9%, respectively; *P* = 0.0004*)*. Among CRAB patients with another Gram-negative species on a clinical culture ± 3 days of the index *A. baumannii* clinical culture, > 80% were identified on the same CRAB culture site. Among patients with a Gram-negative species on a clinical culture ± 3 days of the index *A. baumannii* clinical culture, 28.8% of CRAB patients had a CR Gram-negative pathogen on a clinical site vs. 17.9% of patients with CSAB. The most common Gram-negative pathogens included CR strains of *P. aeruginosa*, *Klebsiella* spp., *Stenotrophomonas maltophilia*, and *E. cloacae*; and the proportions of most CR Gram-negative isolates were significantly higher among patients with CRAB (except CR *E. cloacae*) (Table 2). CR *P. aeruginosa* and CR *K. pneumoniae* were significantly more frequently isolated also from the same culture site with CRAB than with CSAB (Table 2).

**Table 2:** Comparison of microbiologic characteristics between CRAB and CSAB patients at the hospitalization encounter level

Characteristics	CR *A. baumannii*^1^	CS *A. baumannii*^2^	*P* value
Number of encounters	2,708	4,370	
*A. baumannii* on culture at admission (within ± 3 days of admission), n (%)	1,386 (51.2)	2,694 (61.6)	< 0.0001
Hospital days between admission and index *A. baumannii* culture			
Median (IQR)	2.0 (1.0–8.0)	2.0 (1.0–5.0)	< 0.0001
Min–max	0.0–231.0	0.0–224.0	
Patients who were in the ICU on the index *A. baumannii* culture collection day, n (%)	1,165 (43.0)	1,665 (38.1)	< 0.0001
Number of positive *A. baumannii* cultures per hospitalization episode, n (%)			
1	2,143 (79.1)	3,933 (90.0)	< 0.0001
> 1	565 (20.9)	437 (10.0)	
*A. baumannii* culture sites, n (%)			
Single culture site	2,428 (89.7)	4,222 (96.6)	< 0.0001 ^3^
Respiratory	956 (39.4)	1,287 (30.5)	< 0.0001
Wound	829 (34.1)	1,233 (29.2)	
Blood	176 (7.2)	592 (14.0)	
Urine	253 (10.4)	666 (15.8)	
Fluid/serum	36 (1.5)	116 (2.7)	
Lesion/abscess/ulcer	33 (1.4)	59 (1.4)	
Other	145 (6.0)	269 (6.4)	
> 1 culture site	280 (10.3)	148 (3.4)	< 0.0001 ^3^
Blood or respiratory or both involved	210 (75.0)	112 (75.7)	0.0237
Multisite, neither blood nor respiratory involved	70 (25.0)	36 (24.3)	
Presence of other Gram-negative pathogens in any site within ± 3 days of the index *A. baumannii* clinical culture, n (%)	1,279 (47.2)	1,876 (42.9)	0.0004
Presence of other Gram-negative pathogen that are CR in any site within ± 3 days of the index *A. baumannii* clinical culture, n (% of total other Gram-negative pathogens)	369 (28.9)	336 (17.9)	< 0.0001
Presence of other Gram-negative pathogens in the same site as *A. baumannii* within ± 3 days of index *A. baumannii* clinical culture, n (%)	1,071 (39.5)	1,639 (37.5)	0.0856
Presence of other Gram-negative pathogen that are CR in the same site as *A. baumannii* within ± 3 days of the index *A. baumannii* clinical culture (% of total other Gram-negative pathogens)	301 (28.1)	308 (18.8)	< 0.0001
Other Gram-negative pathogens present in any site within ± 3 days of the index *A. baumannii* clinical culture, n (%)			
*P. aeruginosa*	511 (18.9)	415 (9.5)	< 0.0001
CR *P. aeruginosa* (as % of total *P. aeruginosa*)	226 (44.2)	53 (12.8)	< 0.0001
*P. mirabilis*	340 (12.6)	211 (4.8)	< 0.0001
CR *P. mirabilis* (as % of total *P. mirabilis*)	7 (2.1)	2 (0.9)	0.0147
*E. coli*	254 (9.4)	356 (8.1)	0.0724
CR *E. coli* infection (as % of total *E. coli*)	7 (2.8)	3 (0.8)	0.0388
*Klebsiella* spp.	300 (11.1)	433 (9.9)	0.1164
CR *Klebsiella* spp. (as % of total *Klebsiella* spp.)	49 (16.3)	18 (4.2)	< 0.0001
*E. cloacae*	53 (2.0)	298 (6.8)	< 0.0001
CR *E. cloacae* (as % of total *E. cloacae*)	7 (13.2)	15 (5.0)	0.5336
*S. maltophilia*	76 (2.8)	230 (5.3)	< 0.0001
Other Gram-negative pathogens present in the same culture as *A. baumannii*, n (%)			
*P. aeruginosa* in the same site as *A. baumannii*	402 (13.7)	355 (7.5)	< 0.0001
CR *P. aeruginosa* infection in the same site as *A. baumannii* (as % of total *P. aeruginosa*)	179 (44.2)	43 (12.1)	< 0.0001
*P. mirabilis* in the same site as *A. baumannii*	257 (8.7)	167 (3.5)	< 0.0001
CR *P. mirabilis* infection in the same site as *A. baumannii* (as % of total *P. mirabilis*)	4 (1.5)	2 (1.2)	0.1521
*E. coli* in the same site as *A. baumannii*	162 (5.5)	239 (5.0)	0.2800
CR *E. coli* infection (as % of total *E. coli*)	5 (3.0)	2 (0.8)	0.0708
*Klebsiella* spp. in the same site as *A. baumannii*	214 (7.3)	351 (7.4)	0.9059
CR *Klebsiella* spp. infection in the same site as *A. baumannii* (as % of total *Klebsiella* spp.)	37 (17.1)	10 (2.8)	< 0.0001
*E. cloacae* in the same site as *A. baumannii*	37 (1.3)	260 (5.5)	< 0.0001
CR *E. cloacae* in the same site as *A. baumannii* (as % of total *E. cloacae*)	5 (13.5)	13 (5.0)	0.3596
*S. maltophilia* in the same site as *A. baumannii*	68 (2.3)	223 (4.7)	< 0.0001

CR, carbapenem resistant; CS, carbapenem susceptible; ICU, intensive care unit; IQR, inter-quartile range; SD, standard deviation

^1^CR *A. baumannii* includes encounters where all cultures were CR, cultures were a mix of CR and CS, or cultures were a mix of CR and with unknown susceptibility

^2^CS *A. baumannii* includes encounters where all cultures were CS, or cultures were a mix of CS and with unknown susceptibility

^3^*P* value for comparison between single culture site and > 1 culture site

### Patient outcomes on the hospitalization encounter level

Comparison of outcomes between patients with CRAB and CSAB at the hospitalization encounter level is shown in Table 3. A significant difference in the distribution of hospital discharge destinations was observed between CRAB and CSAB patients. Patients with CRAB relative to patients with CSAB were more likely to die during their hospitalization (20.5% vs. 11.3%, respectively), were more likely to be transferred to another healthcare facility (38.6% vs. 22.4%, respectively), and were less likely to be discharged to home (18.0% vs. 47.7%). Patients with CRAB had significantly higher 14- and 30-day in-hospital mortality rates and significantly longer total and infection-associated hospital LOS relative to patients with CSAB. Significantly more patients with CRAB than with CSAB resided in the ICU (62.4% vs. 50.0%, *P* < 0.0001) from index culture collection day to discharge or death. The proportion of patients who were admitted to the ICU on or after the collection day of the index *A. baumannii* culture was also significantly higher for patients with CRAB compared with CSAB (53.7% vs. 45.2%, respectively; *P* < 0.0001). Among CRAB patients who were admitted to the ICU on or after index *A. baumannii* culture day, the median (IQR) ICU infection-associated LOS was 4.0 (2.0–9.0) days. In contrast, the median (IQR) ICU infection-associated LOS among CSAB patients who were admitted to the ICU on or after index *A. baumannii*culture day was 5.0 (2.0–11.0) days. Details for CRAB vs. CSAB infections, stratified by presence of another Gram-negative pathogen in any clinical culture site within ± 3 days of the index *A. baumannii* culture are shown in Supplementary Table 2.

**Table 3:** Comparison of outcomes between patients with CRAB and CSAB at the hospitalization encounter level

Category	CR *A. baumannii*^1^	CS *A. baumannii*^2^	*P* value
**Number of encounters**	2,708	4,370	
**Discharge status**^**3**^, **n (%)**			
Death	555 (20.5)	493 (11.3)	< 0.0001
Discharged to hospice	215 (7.9)	226 (5.2)	
Discharged to home	487 (18.0)	2,085 (47.7)	
Transferred to another healthcare facility	1,045 (38.6)	980 (22.4)	
Other	406 (15.0)	586 (13.4)	
**14-day all-cause mortality**,** n (%)**	438 (16.2)	378 (8.6)	< 0.0001
**30-day all-cause mortality**,** n (%)**	513 (18.9)	462 (10.6)	< 0.0001
**Total LOS**,** days**			
Median (IQR)	12.0 (7.0–22.0)	9.0 (6.0–19.0)	< 0.0001
**Infection-associated LOS**,** days**			
Median (IQR)	9.0 (5.0–15.0)	8.0 (4.0–14.0)	< 0.0001
Min–max	1.0–517.0	1.0–374.0	
**Residence in the ICU from index culture collection day to hospital discharge or death**,** n (%)**	1,691 (62.4)	2,184 (50.0)	< 0.0001
**ICU infection-associated LOS among patients from index culture collection day to hospital discharge or death**,** days**			
Median (IQR)	6.0 (2.0–14.0)	7.0 (2.0–17.0)	0.0005
Min–max	1.0–309.0	1.0–256.0	
**Patients admitted to the ICU on or after the index ** ***A. baumannii*** ** culture collection day,** **n (%)**	1,456 (53.7)	1,977 (45.2)	< 0.0001
**ICU infection-associated LOS among patients admitted to the ICU on or after the index ** ***A. baumannii*** ** culture collection day,** **days**			
Median (IQR)	4.0 (2.0–9.0)	5.0 (2.0–11.0)	0.0208
Min–max	1.0–279.0	1.0–226.0	

CR, carbapenem resistant; CS, carbapenem susceptible; ICU, intensive care unit; IQR, inter-quartile range; IV, intravenous; LOS, length of stay; SD, standard deviation

^1^CR *A. baumannii* includes encounters where all cultures were CR, cultures were a mix of CR and CS, or cultures were a mix of CR and with unknown susceptibility

^2^CS *A. baumannii* includes encounters where all cultures were CS, or cultures were a mix of CS and with unknown susceptibility

^3^Discharge statuses are grouped as follows. Death includes any indication that the patient expired (overall and, for hospice care, expired at home, in a medical facility, or in an unknown place). Discharged to hospice includes both home hospice and medical facility hospice. Discharged to home includes: discharged to home or self-care; discharged to home health organization; discharged to a home IV provider; discharged to home with self-planned acute inpatient readmission; and discharged to home health with a planned acute inpatient readmission. Transfers to another facility include discharges with transfers to any other type of facility excluding home health. Other discharge statuses include: left against medical advice; admitted as an inpatient to this hospital; still a patient and expected to return; discharged to this or another institution for outpatient clinic service; discharged or transferred to the court or law enforcement; or information not available

## Discussion

Despite being recognized a predominant healthcare-associated pathogen and major cause of morbidity, mortality, and healthcare costs [17, 21, 25–27], epidemiology data on *A. baumannii* and CRAB among adult hospitalized patients in the USA remains limited [13–15, 28]. To gain a better understanding of the epidemiology of *A. baumannii* and CRAB among adult patients across US hospitals, we sought to determine the rates of *A. baumannii* and CRAB cases per 100 adult hospitalization encounters in the 338 hospitals that reported clinical and microbiological information in the US PINC AI™ electronic healthcare database between 2018 and 2022. Overall, we found *A. baumannii* was identified on a clinical culture in approximately 1% of the current sample of all adult US hospital admissions, including a total of 613,244 hospitalization encounters over the study period. From a longitudinal perspective, a slight increase in the yearly incidence rates of *A. baumannii* cases per 100 adult hospitalization encounters was observed between 2018 and 2021 but in 2022, the rate dropped below 2019 levels. Although this finding suggests *A. baumannii* incidence rates in the USA have remained somewhat consistent over the study period, the incidence of CRAB cases per 100 adult hospitalization encounters in the US PINC AI™ steadily increased from 0.39 cases per 100 hospitalization encounters in 2018 to 0.53 cases per 100 hospitalization encounters in 2022. More importantly, considerable variability in the CRAB incidence rates was observed across the US census regions, with the highest incidence rates observed in the West South Central, East North Central, and East South Central regions. Across these regions, there were 0.63–0.78 cases per 100 adult hospitalization encounters, equating to one CRAB case per every 120–160 adult hospital admissions in the USA.

The increasing CRAB incidence rate observed in this analysis, especially during the COVID-19 pandemic period, is in contrast with the CRAB incidence rates reported by several groups for previous years [3, 29, 30]. Data from the US CDC showed that the incidence rate of CRAB infections and deaths among hospital-acquired infections decreased between 2012 and 2018 and remained relatively stable until 2020 [3]. Representing a population of over 19 million individuals across 9 states, the overall crude CRAB incidence rate was 0.61/100,000 persons among patients with healthcare-associated infections in 2020 [3]. The CDC noted in their 2020 report that urinary tract infection was the most frequent infection type, although culture collection was restricted to normally sterile sites and excluded the respiratory tract and wounds [3, 28]. However, the incidence rate increased to 1.9/100,000 persons after inclusion of respiratory and skin samples in the CDC analysis, aligning with our results [31]. In our data, CRAB cases were most commonly found also in the respiratory tract and wound cultures, similar to the CDC data from 2021 [31]. Additionally, our finding on the increasing trend in CRAB incidence rates during the study period also align with a recent study from Italy, which reported 7.5- and 5.5-fold increases in CRAB colonization and infection, respectively, in ICUs during the COVID-19 pandemic (January–April 2020) vs. the period of January–April 2019 [32].

Another notable finding in this analysis was the high prevalence (> 45%) of other Gram-negative species in a clinical culture within ± 3 days of the index *A. baumannii* clinical culture. The presence of another Gram-negative pathogen on any clinical culture within ± 3 days of the index *A. baumannii* clinical culture was more common in patients with CRAB (48.6%) compared with patients with CSAB (43.8%). In nearly all CRAB patients (> 85%), the presence of other Gram-negative pathogens was identified in the same culture site as the index CRAB clinical culture. Irrespective of the culture site (i.e., any or same as the index CRAB culture), ~ 30% of other Gram-negative pathogens in the CRAB group were reported to be CR. Consistent with other studies, the most common Gram-negative pathogens reported to be CR among CRAB patients were *P. aeruginosa*, *Klebsiella* spp., *S. maltophilia*, and *E. cloacae* [27, 33, 34]. As might be anticipated, patients with CRAB infections in our analysis tended to be older and have more comorbid conditions than patients with CSAB [11, 20, 21, 30, 35]. Patients with CRAB were also more likely to be in the ICU at index culture collection than with CSAB. While CRAB is considered to predominately cause infections among ICU patients [36], our findings suggest that CRAB is not limited to the ICU and is likely pervasive across all wards in US hospitals [37]. This finding is consistent with a recent multi-center US study of hospitalized patients with Gram-negative infections, which reported that most patients with CR Gram-negative infections, including CRAB, either resided on a non-ICU ward or had a CR Gram-negative infection at admission [38].

The epidemiologic findings from this study have important implications for clinical practice given the high morbidity, mortality, and healthcare costs associated with the management of adult hospitalized patients with infections due to CRAB and CSAB [18, 25, 29, 39–41]. Although the primary objective of this study was elucidation of the epidemiology of *A. baumannii* and CRAB infections in hospitalized patients, our results also align with previous findings showing that patients with CRAB have worse outcomes than patients with CSAB [20, 35]. Given the observed incidence rates of CRAB and CSAB in this study and the critical importance of early appropriate therapy, clinicians should consider *A. baumannii*, especially CRAB, as a potential pathogen in adult hospitalized patients presenting with clinical signs and symptoms of infections. Its relevance should be strongly considered in hospitals with ongoing outbreaks or high rates of CRAB on the local antibiogram. Furthermore, the risk factors for *A. baumannii* and CRAB are well described in the literature (e.g., prior colonization, residence in a non-acute healthcare facility, prior exposure to antibiotics, mechanical ventilation) [18, 35, 42, 43]. Knowledge of these risk factors should be implemented when starting treatment with antibiotics targeting *A. baumannii* or CRAB, including high-dose ampicillin-sulbactam in combination with polymyxins, tigecycline, or cefiderocol or sulbactam-durlobactam in combination with a carbapenem, for “at-risk” patient populations [12, 35, 44]. For patients with well-defined risk factors of CRAB infections, even prior to a confirmed culture result, clinicians should also assess if the patient is at risk for an infection with another CR Gram-negative pathogen, given the high prevalence of patients with CRAB on a clinical culture in this study who had ≥ 1 other Gram-negative pathogen(s) on a clinical culture within ± 3 days of the index *A. baumannii* culture. It is well known that delays in appropriate antimicrobial therapy, post emergence of signs and symptoms of an infection, are associated with worse outcomes in patients with serious Gram-negative bacterial infections [10]. Thus, clinicians may prefer initiating a broader, more aggressive antibiotic therapy in patients at risk of a serious infection caused by *A. baumannii* or CRAB. Rapid diagnostics may help to guide microbiological information more rapidly and clarify the presence of specific carbapenemases associated with *A. baumannii* or other Gram-negative pathogens [45]. Management of patients through utilization of risk stratification tools, routine screening and rapid diagnostics will be critically important for clinicians when considering empirical treatment for patients with *A. baumannii* or CRAB since culture results are typically known after 48–72 h following culture collection [46].

Moving forward, molecular characterization of CRAB isolates may help to understand the virulence and spread of this species in US centers. Recent investigations have shown that the clonal type CC92_OX_ is highly prevalent in the USA, and the most frequently acquired β-lactamase is OXA-23, with a small proportion having intrinsic chromosomal OXA enzymes as well [47, 48]. Increasing trends were observed in carbapenemase-producing CRAB in Alameda County, California, between July 2019 and July 2021 and in a collection of isolates from the Antimicrobial Resistance Laboratory Network (2017–2020) [49, 50]. Understanding the local molecular characteristics of CRAB isolates can help in the selection of antibiotic treatment, as susceptibility phenotypes can vary based on presence or absence of acquired and/or chromosomal OXA enzymes [7, 47, 48]. Current recommendations for selecting the most appropriate antibiotic treatment for CRAB incorporate antibiotic susceptibility rates, pharmacokinetics/ pharmacodynamic concepts, mechanisms of resistance and potential side effects [12]. Although the Infectious Diseases Society of America guidance on treatment of multidrug-resistant and CRAB infections does not address infection control [44], a recent multidisciplinary long-term study has shown that the burden of hospital-acquired CRAB infections can be reduced by means of routine surveillance cultures to detect colonization in asymptomatic carriers, particularly when patients are screened at multiple body sites through collection of skin, pharyngeal, and rectal swabs [51]. Utilizing a screening method with high sensitivity may help to identify colonized patients more rapidly, however, lack of a positive sample may not be considered as evidence of non-carriers in areas with high prevalence [52].

Although this study utilized a large generalizable dataset representative of US hospitals to identify patient characteristics and cases of *A. baumannii*, several limitations can be noted. While the PINC AI™ is largely representative of the US hospitals, the current sample is a convenience (non-random) sample, and the southern portion of the US is overrepresented in the database. This may explain, in part, the observed regional differences in *A. baumannii* and CRAB infection rates. While we limited the study to adult hospitalized patients with *A. baumannii* on a clinical culture, it is possible that some may have been colonized patients vs. infected patients. Thus, the incidence rates reported in this study should not be interpreted as *A. baumannii* and CRAB infection rates. Rather, the incidence rates reflect the number of hospitalized patients with *A. baumannii* and CRAB on a clinical culture per 100 hospital encounters and likely overestimate the true *A. baumannii* and CRAB infection rates across US hospitals. Additionally, we were unable to differentiate between hospital-acquired and community-acquired *A. baumannii* infections due to the limited pre-hospitalization/outpatient data in patients who had a positive *A. baumannii* culture within 3 days of admission. Although data were collected from 2020 to 2021, patients hospitalized with COVID-19 pneumonia were not compared with patients without COVID-19 pneumonia or infection. It is assumed that all-cause mortality rates were affected by timeliness of appropriate treatment and management of COVID-19 in 2020 and 2021, both of which were not collected in this study [53]. The role of inappropriate and/or delayed antibiotic treatment was not evaluated in this analysis due to the descriptive nature of the outcome analyses. The outcome analyses were included to provide a crude mortality rate in patients with CRAB and CSAB on a clinical culture. Additionally, mortality may be underestimated in the present analysis because only in-hospital mortality could be analyzed for this patient population based on the database and because there is no link to the national death index. Further analyses are still required to accurately describe the clinical and economic burden associated with CRAB hospitalizations among adult patients across US hospitals. Lastly, the study relied on phenotypic data for identifying CRAB and no genotypic data were available within PINC AI™. Unfortunately, the hospitals within PINC AI™ are de-identified and we are unable to ascertain any additional information on susceptibility data across institutions. We were also unable to examine more clinically relevant outcomes like emergence of resistance to the newer agents during therapy given the limited susceptibility data on newer agents in PINC AI™. As such, there is a need for future, large-scale studies to determine the impact of carbapenem resistance on the outcomes of patients with *A. baumannii*. Future studies should include, when possible, complete antibiotic susceptibility, treatment, and genotypic data when characterizing the epidemiology and outcomes of CRAB across hospitals over time.

## Conclusions

In this retrospective, multicenter study of adult hospitalized patients in the PINC AI™ database, *A. baumannii* was recovered on a clinical culture in 1% of hospitalized US patients. While the results suggest *A. baumannii* incidence rates in the USA have remained somewhat consistent over the 4-year study period, the incidence of CRAB cases per 100 adult hospitalization encounters steadily increased between 2018 and 2022 and there was considerable variability in the CRAB incidence rates across the US census regions. A high prevalence (> 43%) of other Gram-negative pathogens in a clinical culture within ± 3 days of the index *A. baumannii* clinical culture was observed among both CRAB and CSAB patients and ~ 30% of other Gram-negative pathogens in the CRAB group were reported to be CR. In concordance with previous reports, patients with CRAB relative to patients with CSAB had increased mortality, longer hospital LOS, and lower probability of being discharged home. Given the high incidence rates of CRAB and CSAB observed in this study and critical importance of early appropriate therapy, clinicians should consider *A. baumannii*, especially CRAB, as a potential pathogen in adult hospitalized patients presenting with a clinical symptom consistent with *A. baumannii* infection. As patients with CRAB infections currently have very limited treatment options, commonly relying mainly on older antibiotics, newer agents are urgently needed. Implementation of strict infection control and routine surveillance may help to reduce the burden.

## Electronic supplementary material

Below is the link to the electronic supplementary material.

Supplementary Material 1

Supplementary Material 2

## Data Availability

This study is conducted using an anonymous, commercially available secondary healthcare database called PINC AI^TM^ Database that meets the US HIPAA requirement. The data is not sharable per our license agreement with the data owner.

## Abbreviations

CCI: Charlson Comorbidity Index
CDC: US Centers for Disease Control and Prevention
COVID-19: coronavirus disease 2019
CR: carbapenem resistant
CRAB: carbapenem-resistant *Acinetobacter baumannii*
CS: carbapenem susceptible
CSAB: carbapenem-susceptible *Acinetobacter baumannii*
ICU: intensive care unit
IQR: interquartile range
LOS: length of stay
OXA: oxacillinase
WHO: World Health Organization
