# Supplementary material for: Clinical burden of Acinetobacter baumannii, including carbapenem-resistant A. baumannii, in hospitalized adult patients in the USA between 2018 and 2022
Source: BMC Infect Dis. 2025 Apr 17;25:549. doi: 10.1186/s12879-025-10749-1 (PMC12004818; doi:10.1186/s12879-025-10749-1)
Supplement: Supplementary file 1 — Supplementary Material 1 [file 12879_2025_10749_MOESM1_ESM.docx]

**SUPPLEMENATARY MATERIAL**

**Additional File 1**

**Supplementary Table 1** Hospitalization encounters with *Acinetobacter baumannii* culture between January 1, 2018 and December 31, 2022

| **Year** | **Category** | **Number of Hospitalization Encounters** | **Rate per 100 Hospitalization Encounters** | **Number of Hospitalized Patients** | **Rate per 100 Hospitalized Patients** |
| --- | --- | --- | --- | --- | --- |
| **Overall** | All hospitalization encounters with microbiology data | 613,244 |  | 514,975 |  |
|  | Number of hospitalization encounters with ≥1 *A. baumannii* on clinical culture | 7,270 | 1.19 | 6,842 | 1.33 |
|  | - Absence of a Gram-negative pathogen in any clinical culture site within ±3 days of the index *A. baumannii* culture | 3,991 | 0.65 | 3,855 | 0.75 |
|  | - Presence of a Gram-negative pathogen in any clinical culture site within ±3 days of the index *A. baumannii* culture | 3,279 | 0.53 | 3,138 | 0.61 |
|  | - Absence of a Gram-negative pathogen in same clinical culture within ±3 days of the index *A. baumannii* culture | 4,448 | 0.73 | 4,287 | 0.83 |
|  | - Presence of a Gram-negative pathogen in same clinical culture site within ±3 days of the index *A. baumannii* culture | 2,822 | 0.46 | 2,711 | 0.53 |
| **2018** | All hospitalization encounters with microbiology data | 149,817 |  | 134,749 |  |
|  | Number of hospitalization encounters with ≥1 *A. baumannii* on clinical culture | 1,719 | 1.15 | 1,640 | 1.22 |
|  | - Absence of a Gram-negative pathogen in any clinical culture within ±3 days of the index *A. baumannii* culture | 964 | 0.64 | 934 | 0.69 |
|  | - Presence of a Gram-negative pathogen in any clinical culture site within ±3 days of the index *A. baumannii* culture | 755 | 0.50 | 740 | 0.55 |
|  | - Absence of a Gram-negative pathogen in same clinical culture within ±3 days of the index *A. baumannii* culture | 1,074 | 0.72 | 1,039 | 0.77 |
|  | - Presence of a Gram-negative pathogen in same clinical culture site within ±3 days of the index *A. baumannii* culture | 645 | 0.43 | 634 | 0.47 |
| **2019** | All hospitalization encounters with microbiology data | 143,256 |  | 129,342 |  |
|  | Number of hospitalization encounters with ≥1 *A. baumannii* on clinical culture | 1,640 | 1.14 | 1,554 | 1.20 |
|  | - Absence of a Gram-negative pathogen in any clinical culture within ±3 days of the index *A. baumannii* culture | 899 | 0.63 | 866 | 0.67 |
|  | - Presence of a Gram-negative pathogen in any clinical culture site within ±3 days of the index *A. baumannii* culture | 741 | 0.52 | 715 | 0.55 |
|  | - Absence of a Gram-negative pathogen in same clinical culture within ±3 days of the index *A. baumannii* culture | 1,000 | 0.70 | 963 | 0.74 |
|  | - Presence of a Gram-negative pathogen in same clinical culture site within ±3 days of the index *A. baumannii* culture | 640 | 0.45 | 620 | 0.48 |
| **2020** | All hospitalization encounters with microbiology data | 133,453 |  | 121,421 |  |
|  | Number of hospitalization encounters with ≥1 *A. baumannii* on clinical culture | 1,663 | 1.25 | 1,600 | 1.32 |
|  | - Absence of a Gram-negative pathogen in any clinical culture within ±3 days of the index *A. baumannii* culture | 926 | 0.69 | 910 | 0.75 |
|  | - Presence of a Gram-negative pathogen in any clinical culture site within ±3 days of the index *A. baumannii* culture | 737 | 0.55 | 716 | 0.59 |
|  | - Same-site monomicrobial^c^ | 1,023 | 0.77 | 1,005 | 0.83 |
|  | - Presence of a Gram-negative pathogen in same clinical culture site within ±3 days of the index *A. baumannii* culture | 640 | 0.48 | 621 | 0.51 |
| **2021** | All hospitalization encounters with microbiology data | 120,348 |  | 110,126 |  |
|  | Number of hospitalization encounters with ≥1 *A. baumannii* on clinical culture | 1,508 | 1.25 | 1,466 | 1.33 |
|  | - Absence of a Gram-negative pathogen in any clinical culture within ±3 days of the index *A. baumannii* culture | 828 | 0.69 | 813 | 0.74 |
|  | - Presence of a Gram-negative pathogen in any clinical culture site within ±3 days of the index *A. baumannii* culture | 680 | 0.57 | 666 | 0.60 |
|  | - Absence of a Gram-negative pathogen in same clinical culture within ±3 days of the index *A. baumannii* culture | 924 | 0.77 | 908 | 0.82 |
|  | - Presence of a Gram-negative pathogen in same clinical culture site within ±3 days of the index *A. baumannii* culture | 584 | 0.49 | 575 | 0.52 |
| **2022** | All hospitalization encounters with microbiology data | 66,370 |  | 60,851 |  |
|  | Number of hospitalization encounters with ≥1 *A. baumannii* on clinical culture | 740 | 1.11 | 712 | 1.17 |
|  | - Absence of a Gram-negative pathogen in any clinical culture within ±3 days of the index *A. baumannii* culture | 374 | 0.56 | 369 | 0.61 |
|  | - Presence of a Gram-negative pathogen in any clinical culture site within ±3 days of the index *A. baumannii* culture | 366 | 0.55 | 353 | 0.58 |
|  | - Absence of a Gram-negative pathogen in same clinical culture within ±3 days of the index *A. baumannii* culture | 427 | 0.64 | 419 | 0.69 |
|  | - Presence of a Gram-negative pathogen in same clinical culture site within ±3 days of the index *A. baumannii* culture | 313 | 0.47 | 305 | 0.50 |
