# Supplementary material for: Clinical burden of Acinetobacter baumannii, including carbapenem-resistant A. baumannii, in hospitalized adult patients in the USA between 2018 and 2022
Source: BMC Infect Dis. 2025 Apr 17;25:549. doi: 10.1186/s12879-025-10749-1 (PMC12004818; doi:10.1186/s12879-025-10749-1)
Supplement: Supplementary file 2 — Supplementary Material 2 [file 12879_2025_10749_MOESM2_ESM.docx]

**SUPPLEMENATARY MATERIAL**

**Additional File 2**

**Supplementary** **Table 2** Outcomes in patients with positive *Acinetobacter baumannii* infections according to carbapenem susceptibility

|  | Absence of a Gram-negative pathogen in any site within ±3 days of the index *A. baumannii* culture | | | Presence of ≥ 1 Gram-negative pathogen in any site within ±3 days of the index *A. baumannii* culture | | |
| --- | --- | --- | --- | --- | --- | --- |
| **Category** | **CR *A. baumannii*** | **CS *A. baumannii*** | ***P* value** | **CR *A. baumannii*** | **CS *A. baumannii*** | ***P* value** |
| **Total N** | 1,637 | 2,731 |  | 1,071 | 1,639 |  |
| **Discharge status^a^, n (%)** |  |  |  |  |  |  |
| Death | 389 (23.8) | 329 (12.0) | <0.0001 | 166 (15.5) | 164 (10.0) | <0.0001 |
| Discharged to hospice | 132 (8.1) | 143 (5.2) |  | 83 (7.7) | 83 (5.1) |  |
| Discharged to home | 304 (18.6) | 1,336 (48.9) |  | 183 (17.1) | 749 (45.7) |  |
| Transferred to another healthcare facility | 577 (35.2) | 568 (20.8) |  | 468 (43.7) | 412 (25.1) |  |
| Other | 235 (14.4) | 355 (13.0) |  | 171 (16.0) | 231 (14.1) |  |
| 14-day all-cause mortality after index culture, n (%) | 318 (19.4) | 256 (9.4) | <0.0001 | 120 (11.2) | 122 (7.4) | 0.0008 |
| 30-day all-cause mortality after index culture, n (%) | 366 (22.4) | 308 (11.3) | <0.0001 | 147 (13.7) | 154 (9.5) | 0.0005 |
| **LOS, days** |  |  |  |  |  |  |
| Median (IQR) | 13.0 (7.0–22.0) | 9.0 (5.0–18.0) | <0.0001 | 12.0 (8.0–22.0) | 10.0 (6.0–20.0) | <0.0001 |
| Min–Max | 2.0–519.0 | 2.0–411.0 |  | 2.0–310.0 | 2.0–475.0 |  |
| **Infection-associated LOS, days** |  |  |  |  |  |  |
| Median (IQR) | 9.0 (5.0–14.0) | 7.0 (4.0–13.0) | <0.0001 | 9.0 (5.0–16.0) | 8.0 (5.0–15.0) | 0.002 |
| Min–Max | 1.0–517.0 | 1.0–374.0 |  | 1.0–280.0 | 1.0–323.0 |  |
| **Residence in an ICU from index culture collection day to hospital discharge or death, n (%)** | 1,043 (63.7) | 1,373 (50.3) | <0.0001 | 648 (60.5) | 811 (49.5) | <0.0001 |
| **ICU infection-associated LOS among patients from index culture collection day to hospital discharge or death, days** |  |  |  |  |  |  |
| Median (IQR) | 6.0 (2.0–15.0) | 7.0 (2.0–17.0) | 0.269 | 5.0 (2.0–13.0) | 8.0 (3.0–18.0) | <0.0001 |
| Min–Max | 1.0–159.0 | 1.0–256.0 |  | 1.0–309.0 | 1.0–166.0 |  |
| **Patients admitted to the ICU on or after the index *A. baumannii* culture collection day, n (%)** | 902 (86.5) | 1,235 (90.0) | <0.0001 | 554 (85.5) | 742 (91.5) | 0.001 |
| **ICU infection-associated LOS among patients admitted to the ICU on or after the index *A. baumannii* culture collection day, days** |  |  |  |  |  |  |
| Median (IQR) | 4.0 (2.0–9.0) | 4.0 (2.0–11.0) | 0.360 | 4.0 (2.0–9.0) | 5.0 (2.0–12.0) | 0.010 |
| Min–max | 1.0–90.0 | 1.0–226.0 |  | 1.0–279.0 | 1.0–166.0 |  |

CR, carbapenem resistant; CS, carbapenem susceptible; ICU, intensive care unit; IQR, inter-quartile range; IV, intravenous; LOS, length of stay; SD, standard deviation.

^a^Discharge statuses are grouped as follows. Death includes any indication that the patient expired (overall and, for hospice care, expired at home, in a medical facility, or in an unknown place). Discharged to hospice includes both home hospice and medical facility hospice. Discharged to home includes: discharged to home or self-care; discharged to home health organization; discharged to a home IV provider; discharged to home with self-planned acute inpatient readmission; and discharged to home health with a planned acute inpatient readmission. Transfers to another facility include discharges with transfers to any other type of facility excluding home health. Other discharge statuses include: left against medical advice; admitted as an inpatient to this hospital; still a patient and expected to return; discharged to this or another institution for outpatient clinic service; discharged or transferred to the court or law enforcement; or information not available.
